# Abnormal Resting-State Functional Connectivity of Insular Subregions and Disrupted Correlation with Working Memory in Adults with Attention Deficit/Hyperactivity Disorder

**DOI:** 10.3389/fpsyt.2017.00200

**Published:** 2017-10-11

**Authors:** Qihua Zhao, Hui Li, Xiaoyan Yu, Fang Huang, Yanfei Wang, Lu Liu, Qingjiu Cao, Qiujin Qian, Yufeng Zang, Li Sun, Yufeng Wang

**Affiliations:** ^1^Peking University Sixth Hospital, Institute of Mental Health, Beijing, China; ^2^National Clinical Research Center for Mental Disorders, Key Laboratory of Mental Health, Ministry of Health, Peking University, Beijing, China; ^3^Center for Cognition and Brain Disorders, Hangzhou Normal University, Hangzhou, China; ^4^Zhejiang Key Laboratory for Research in Assessment of Cognitive Impairments, Hangzhou, China

**Keywords:** attention deficit/hyperactivity disorder, insular subregions, resting-state functional connectivity, executive function, salience network, default mode network

## Abstract

**Objectives:**

Executive function (EF) deficits are major impairments in adults with attention deficit/hyperactivity disorder (ADHD). Previous studies have shown that the insula is involved in cognitive and EFs. However, the insula is highly heterogeneous in function, and few studies have focused on functional networks which related to specific insular subregions in adults with ADHD. We explored the functional networks of the insular subregions [anterior insula (AI), mid-insula (MI), and posterior insula (PI)]. Furthermore, their correlations with self-ratings of ecological EFs, including inhibition, shifting, and working memory were investigated.

**Methods:**

Resting-state functional magnetic resonance imaging data in 28 adults with ADHD and 30 matched healthy controls (HCs) were analyzed. The seed-based resting-state functional connectivity (RSFC) of the insular subregions was evaluated. We also investigated their associations with the Behavior Rating Inventory of Executive Function-Adult Version (BRIEF-A) inhibition, working memory, and shifting factor scores.

**Results:**

Compared with HCs, adults with ADHD showed altered RSFC of the AI, with the precuneus, precentral gyrus, and inferior temporal gyrus extended to the middle temporal gyrus, lingual gyrus, and superior occipital gyrus, respectively. There were no significant differences in RSFC of the MI and PI between the two groups. Within the HC group, working memory scores were associated with the RSFC of AI with precuneus and temporal gyrus. However, there was no correlation between these variables in the ADHD group.

**Conclusion:**

The study evaluated RSFC patterns of the insular subregions in adults with ADHD for the first time. Altered RSFC of the AI which is a crucial region of salience network (SN) and part of regions in default mode network (DMN), were detected in adults with ADHD in both results with and without global signal regression (GSR), suggesting that disrupted SN-DMN functional connectivity may be involved in EF impairments in adults with ADHD, especially with respect to working memory. Deficits of the AI which is involved in salient stimuli allocation, might be associated with the pathophysiology of ADHD. The inconsistent results of MI and PI between analyses with and without GSR need further exploration.

## Introduction

Attention deficit/hyperactivity disorder (ADHD) is a childhood-onset neurodevelopmental disorder characterized by developmentally inappropriate symptoms of inattention, hyperactivity, and impulsivity (1). ADHD has an estimated worldwide prevalence of 7.2% among children (2) and in approximately 70% of those diagnosed in childhood, the condition persists into adulthood (3). The persistence of ADHD into adulthood is significantly associated with comorbid anxiety, depression, substance dependence, and emotional dysregulation, exerting effects on the individual’s quality of life (4, 5).

Executive function (EF) deficits are major impairments in ADHD. They include inhibition, working memory, shifting, and so on (6–10). On the basis of a recent review, the core EFs include inhibition, working memory, and cognitive flexibility [also called set shifting (11)]. Furthermore, higher-order EFs, such as reasoning, problem solving, and planning, are built on the foundation of the three core EFs (11). Ecological EF may be more predictive of occupational function than the cognitive performance test (12). Hence, we chose the three factors from the Behavior Rating Inventory Executive Function—Adult Version [BRIEF-A (13)], which is used to estimate ecological EFs.

The pathophysiology of EF impairments in patients with ADHD is still unclear. Convergent evidence indicated that EF impairment in ADHD was associated with functional and structural abnormalities in large-scale brain networks consisting of the prefrontal cortex, parietal lobe, occipital cortex, temporal lobe, insula, and limbic regions (14). Recently, Menon hypothesized the triple network model which consists of the central executive network (CEN), salience network (SN), and default mode network [DMN (15)]. The model indicated that abnormalities in the engagement and disentanglement of the three networks play an important role in many psychiatric disorders, such as depression, autism, and schizophrenia (15). Abnormal cognitive control and state switching in ADHD were also found to be related to aberrant connectivity within SN, CEN, DMN, and attention networks (10, 16). Among these brain networks and regions underlying the neuropathologic mechanism of ADHD, the insula is a critical part of SN and involved in multiple dimensions of EF (17–21). The insula plays an important role in cognitive and affective processes (22). Previous studies have found structural and functional abnormalities in the insula in patients with ADHD. Lopez-Larson et al. revealed a reduction in gray matter volume of the anterior part of the bilateral insula in children with ADHD and that the volume of the right anterior insula (AI) was associated with attention problems and inhibition (19). The insula displayed a significantly abnormal activation during various cognitive tasks including during attention (17, 18), decision making (23), state switching (10), inhibition (24, 25), and working memory tasks (26) in patients with ADHD. In addition, recent studies indicated that children with ADHD showed a decreased functional connectivity between the insula and amygdala interacting with emotional regulation (27, 28).

Previous studies have investigated the abnormal function of the entire insula in patients with ADHD. However, the insula is a complicated and functionally heterogeneous structure. Based on previous studies on insular structure and function (29–31), the insula can be partitioned into three major subregions: the AI, mid-insula (MI), and posterior insula (PI). The AI is implicated in cognitive processes such as working memory, decision making, state switching (shifting), socioemotional function, and interoception (32–34). Adults with ADHD also showed impairments in these processes. The MI is correlated with reactions to olfactogustatory stimuli, sensorimotor function, the awareness of body movement (34), and socioemotional functions. The PI is also implicated in sensorimotor function and interoception (34, 35). The aberrant function and structure of the insula have been investigated in patients with psychiatric diseases such as schizophrenia (36–38), mood disorder (39–42), autism spectrum disorder (43), and posttraumatic stress disorder (44, 45). Different insular subregions have different functions, and a systematic exploration of the neural functional connectivity of insular subregions in patients with ADHD has yet to be conducted.

To explore the neural networks alterations of the insular subregions and the mechanism correlated with ecological EF from a broader view, the present study utilized the seed-based resting-state functional connectivity (RSFC) to investigate the functional connectivity patterns of AI, MI, and PI. Then, we explored their correlations with ecological EFs, including inhibition, working memory and shifting factor scores. Based on prior literature, we expected that (a) patients with ADHD compared with healthy controls (HCs) would show significantly altered RSFC between the insular subregions and other regions (especially ACC and regions within CEN and DMN) and (b) altered RSFC in adults with ADHD between the insular subregions and other brain regions would be correlated with inhibition, working memory, and shifting factor scores.

## Materials and Methods

### Participants

Thirty-five right-handed adults with ADHD were recruited from clinics of Peking University Sixth Hospital and advertisements on the Internet when taking part in cognitive behavioral therapy studies and 30 HCs matched for sex, age, and IQ were enrolled in the study. All of the participants were interviewed, underwent diagnosis and were screened for any potential comorbidities according to exclusion criteria using the Structured Clinical Interview for DSM-IV Axis I Disorders [SCID-I (46)] by a qualified psychiatrist. Conner’s Adult ADHD Diagnostic Interview for DSM-IV (47) was also completed to confirm the diagnosis of ADHD in subjects in the ADHD group. Full-scale IQ measurements were made using the Wechsler Adult Intelligence Scale, Third Edition. All participants met the following criteria: (a) right-handed, (b) no history of head trauma with a loss of consciousness, (c) no history of neurological illness or other severe disease, and (d) no current diagnosis of schizophrenia, severe major depression, clinically significant panic disorder, bipolar disorder, pervasive developmental disorders, or mental retardation (seven participants in the ADHD group were excluded after this review, of whom one had social phobias, one had anorexia nervoasa, two had major depressive disorder, and three had dysthymia disorder); (e) no excessive head movements (>3.0 mm of translation or degrees of rotation in any direction) and (f) a full-scale IQ above 80. A previous or current history of psychiatric disorders, as evidenced in the SCID-I assessment, or neurological disorders resulted in exclusion from the HC groups. Among the 28 adults with ADHD chosen for the final imaging analyses, 21 met the criteria of the inattentive subtype (ADHD-I) and 7 corresponded to the combined subtype (ADHD-C).

Six participants with ADHD had a history of methylphenidate hydrochloride or tomoxetine hydrochloride treatment. Three of the six had stopped the treatment for a long time or had only taken medicine for a short period of time (no >2 months). The other three participants were still being treated with methylphenidate hydrochloride and were required to undergo a washout period of 24 h before the MRI scan. Furthermore, we compared the mean functional connectivity *z*-scores of the clusters, which showed differences between the ADHD and HC groups for patients without history, patients with the history and the HCs. There was no difference in mean functional connectivity *z*-scores of 8 clusters between the two ADHD groups. Furthermore, differences were found between patients with a history and the HCs as well as between patients without a history and the HCs. Therefore, we combined patients with and without medication together to compare with the HC for our final results.

Self-administered scales were completed by all of the participants for diagnostic and correlational purposes.

The ADHD Rating Scale-IV [ADHD RS-IV (48)], a 4-point severity scale (scores 1 indicates no ADHD symptoms, 2 indicates symptoms sometimes, 3 indicates symptoms usually, and 4 indicates symptoms always) was also completed by the participants for the evaluation of the severity of ADHD symptoms. This scale contains 18 items that are consistent with the 18 symptoms in DSM, which consists of 9 inattention items and 9 hyperactivity and impulsivity items.

The Mandarin version of the BRIEF-A consists of 75 items based on the EFs (13). The participants were required to assess their performance in daily life on a 3-point Likert scale (1 equals never; 2 equals sometimes; and 3 equals often). A higher score indicates more EF problems in daily life. The BRIEF-A includes nine factors of EFs: initiating, working memory, planning/organizing, organization of material, task-monitoring, self-monitoring, inhibition, shifting, and emotional control. We chose the working memory, shifting, and inhibition factors from the BRIEF-A. Twenty-seven HCs and 25 patients of the ADHD group completed the BRIEF-A.

This study was approved by the Research Ethics Review Board of Peking University Sixth Hospital. All subjects were fully informed about the research before being asked to sign the informed consent form.

### MRI Data Acquisition

Magnetic resonance imaging data were acquired using a Siemens Trio 3 T scanner (Siemens, Erlangen, Germany) at the Imaging Center for Brain Research at Beijing Normal University. During the resting-state functional magnetic resonance imaging (rs-fMRI) scanning, participants lay in the supine position and were instructed to remain still and relaxed with their eyes closed and to keep their mind vacant but without falling asleep. A head strap and foam pads were used to minimize head movements. Functional images were acquired using an echo-planar imaging sequence with the following parameters: repetition time (TR) = 2,000 ms, echo time (TE) = 30 ms, flip angle = 90°, thickness/skip = 3.5/0.7 mm, matrix = 64 × 64, field of view (FOV) = 200 mm × 200 mm, 33 axial slices, and 240 volumes. High-resolution T1-weighted anatomical images were acquired with the following parameters: TR = 2,530 ms, TE = 3.39 ms, inversion time = 1,100 ms, flip angle = 7°, 128 slices, slice thickness = 1.33 mm, FOV = 256 mm × 256 mm, and matrix = 256 × 256.

### Data Preprocessing

Resting-state fMRI data preprocessing was performed using the Data Processing & Analysis for (Resting-State) Brain Imaging (DPABI) (49). First, we discarded the first 10 volumes. Then, the data were corrected for slice timing and realigned for head motion correction. Next, the structural images were coregistered to the mean functional image. The high-resolution individual T1-weighted images were segmented into gray matter, white matter and cerebrospinal fluid (CSF) by using the “New Segment” method. Subsequently, we performed nuisance signal regression (including 12 derivative motion parameters, global mean signals, white matter, and CSF) to remove the confounding artifacts of head motion and physiological noise (i.e., cardiac and respiratory fluctuations) during the resting state. The functional data were then normalized to the standard Montreal Neurological Institute (MNI) space (resampled voxel size = 3 mm × 3 mm × 3 mm) and spatially smoothed by Diffeomorphic Anatomical Registration Through Exponentiated Lie Algebra (50). The Gaussian kernel full width at half-maximum was 6 mm^3^. Linear detrending of the time series and temporal bandpass filtering (0.01–0.08 Hz) were conducted. Whether preprocessing should include global signal regression (GSR) is controversial (51–53). We conducted an analysis without the GSR, and the results are shown in the supplementary materials (Table S1 and Figures S2–S4 in Supplementary Material).

### Resting-State Functional Connectivity

We selected six spherical seed regions of interest (ROIs) with a radius of 6 mm centered on six MNI coordinates, which were selected based on the previous anatomical and MRI studies (31, 54): the bilateral AI [MNI (*x, y, z*): left = −32, 16, 6; right = 32, 16, 6], the bilateral MI (MNI: left = −38, 2, 8; right = 38, 2, 8), and the bilateral PI (MNI: left = −39, −15, 1; right = 39, −15, 8). The time course of the ROIs were correlated against all other voxels within the whole brain. Individual RSFC maps of the AI, MI and PI were generated by calculating Pearson’s correlation coefficients between the mean time series of the ROIs and the time series of each voxel in the whole brain. Subject-level correlation maps were then converted to *z*-value maps using Fisher’s transformation to improve the normality. All figures were displayed using BrainNet Viewer (55). We used the insular subregion masks (*k* = 3) for further analysis to confirm our results (56). The results comparing the insular subregion masks and spherical ROIs were displayed in the supplementary materials (Figure S5 and Table S2 in Supplementary Material). The results of the differences were very similar to our results.

### Head Motion

Head motion was defined by the mean frame-wise displacement (FD), as described Jenkinson et al. (57). The mean FD (Jenkinson) was calculated to estimate the voxel-wise motion differences between the two groups. The ADHD group and HC group did not differ significantly in the mean FD (*p* = 0.705).

### Statistical Analysis

#### Within-Group Differences in the Connectivity of Insular Subregions

One-sample *t*-tests were performed on *z*-maps of each ROI to generated maps showing significant positive and negative RSFC by applying a gray matter mask implemented in DPABI template within each insular subregions in the adults with ADHD and the HC. A *p* < 0.05 uncorrected indicated that the voxel was significantly correlated with the ROIs. Furthermore, the regions with significant correlations with the ROIs in the two groups were combined to produce a mask for the next analysis.

#### Between-Group Differences in the Connectivity of the Insular Subregions

Two-sample *t*-tests were conducted between the ADHD and HC groups by applying masks which obtained from the significant regions of the combined one-sample *t*-test significant regions. Because of the exploratory nature, the significance of the statistical functional connectivity maps was *p* < 0.05 multiple comparisons using the voxel-wise non-parametric permutation test (5,000 permutations, no acceleration method) with threshold-free cluster enhancement [TFCE (58)] and family wise error corrected. Furthermore, the cluster size of no fewer than 40 voxels. TFCE is an approach for defining a cluster-like voxel-wise statistic in a way that is more natural and stable than the commonly used approach of an initial cluster-forming hard thresholding and gives generally better sensitivity than other methods (58). Besides, non-parametric permutation test is found to produce nominal results than other (59).

### Correlation Calculation

Pearson’s correlation analyses were performed between the RSFC *z*-values of brain regions showing significant group differences and behavioral performances (working memory, inhibition, and shifting factor scores from the BRIEF) in the ADHD and HC groups. Owing to the exploratory nature of these correlation analyses, the statistical level of significance was set at *p* < 0.05, uncorrected.

## Results

### Demographic and Clinical Information

The demographics and clinical characteristics of the ADHD and HC groups were given in Table 1. The adults with ADHD showed significantly higher ADHD RS-IV scores and working memory, inhibition, and shifting factor scores than the adults in the HC group. No differences in sex, age, IQ, and mean FD were detected between the two groups.

**Table 1:** Demographic and clinical information.

Variables	HC (*n* = 30) (mean ± SD)	ADHD (*n* = 28) (mean ± SD)	*T*-Value	*p*-Value
Age (years)	25.92 ± 3.77	27.07 ± 5.48	0.932	0.356
Sex (M/F)	17/13	15/13		0.813
Mean FD (mm)	0.0642 ± 0.0331	0.0608 ± 0.0336	−0.381	0.705
Full-scale IQ scores	123.13 ± 7.12	123.61 ± 9.71	0.212	0.833
ADHD symptoms				
Inattention	12.94 ± 2.21	26.61 ± 3.83	16.488	<0.001
Hyperactivity/impulsivity	12.47 ± 2.50	19.07 ± 4.53	6.808	<0.001
Total scores	25.43 ± 4.03	45.67 ± 6.25	14.550	<0.001
Inhibition factor scores	10.00 ± 1.69 (27)	16.60 ± 2.58 (25)	10.992	<0.001
Working memory factor scores	10.11 ± 1.99 (27)	18.96 ± 2.35 (25)	14.686	<0.001
Shifting factor scores	7.07 ± 1.54 (27)	12.48 ± 2.99 (25)	8.102	<0.001

*HC, healthy control; FD, frame-wise displacement*.

### Functional MRI Results

**Figure 1:**
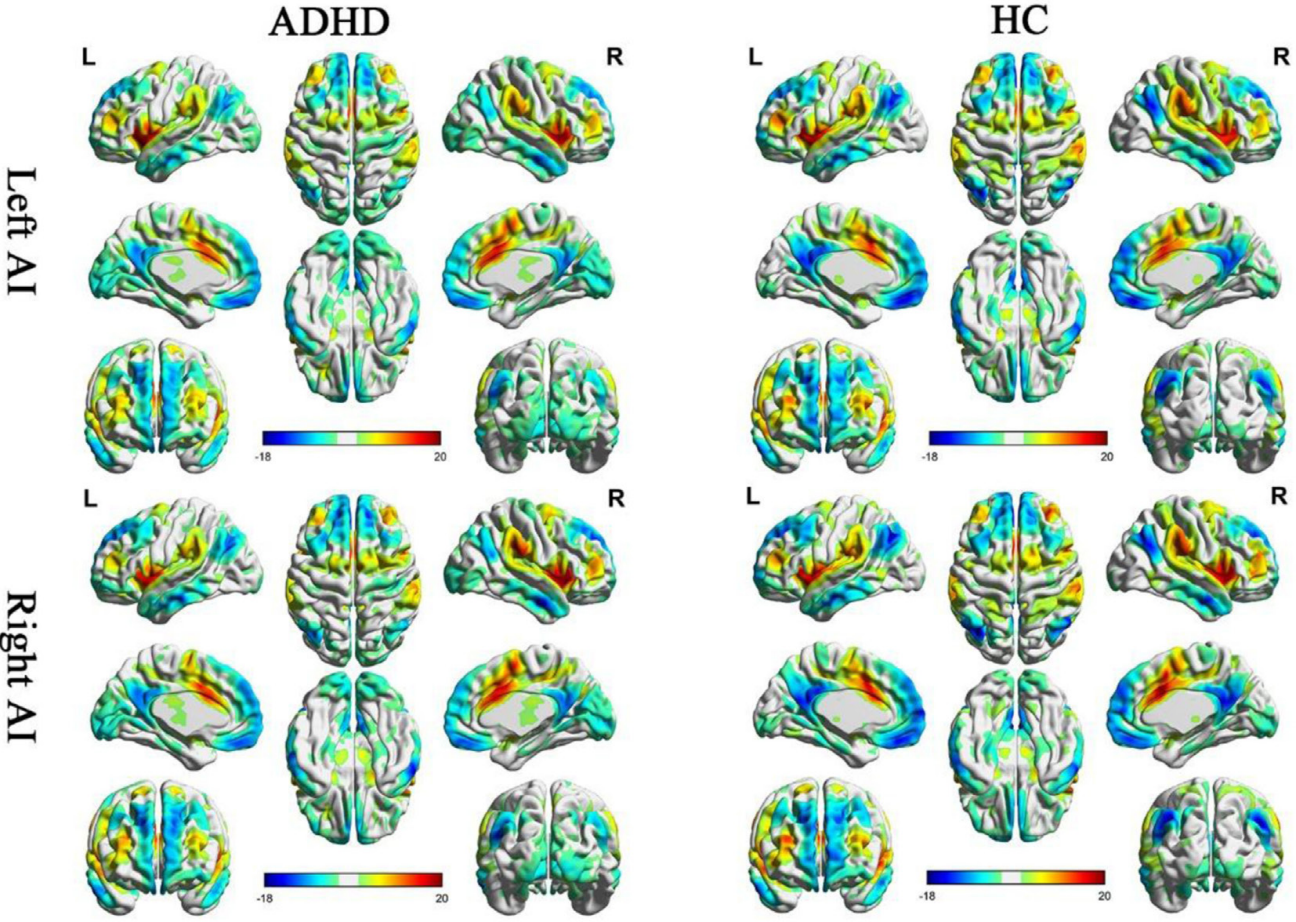
Significant resting-state functional connectivity (RSFC) patterns of the bilateral anterior insula of the attention deficit/hyperactivity disorder (ADHD) and healthy control (HC) groups. The yellow-red colors indicate that the brain regions were positively correlated with the insular subregions and the blue colors indicate that the brain regions were negatively correlated with insular subregions.

One sample *t*-tests displayed the within-group functional connectivity patterns in the ADHD and HC group (Figure 1; Figure S1 in Supplementary Material, respectively). Within the HC group, the functional connectivity patterns showed similar activity in the right and left hemisphere. A significantly positive functional connectivity with the bilateral AI was shown in the partial cortex, temporal lobe, and superior frontal gyrus. A significant negative functional connectivity with the bilateral AI was located in the occipital gyrus, temporal lobe and prefrontal cortex and limbic lobes. Adults with ADHD showed similar RSFC patterns of the bilateral MI and PI as the HCs.

### Between-Group Differences in the RSFC of the Insular Subregions

#### Anterior Insula (Table 2)

**Table 2:** Clusters displaying significant group differences in the RSFC of AI between the ADHD and HC groups.

Seed	Area	L/R	Cluster size	MNI			*t*-Value
				*x*	*y*	*z*	
**Left AI**							
	Inferior temporal gyrus	R	225	57	−66	−3	−4.300
	Lingual gyrus	L	44	−18	−63	−9	−3.919
	Precuneus	R	40	18	−60	27	4.779
	Superior occipital gyrus	L	63	−18	−99	24	−3.984
	Precentral gyrus	R	41	48	−15	51	−4.860
**Right AI**							
	Middle temporal gyrus	R	401	54	−69	3	−4.058
	Lingual gyrus	L	108	−15	−63	−9	−4.309
	Cuneus	L	151	−6	−93	18	−3.618

*MNI, Montreal Neurological Institute; AI, anterior insula; MI, mid-insula; L, left; R, right; HC, healthy control*.

**Figure 2:**
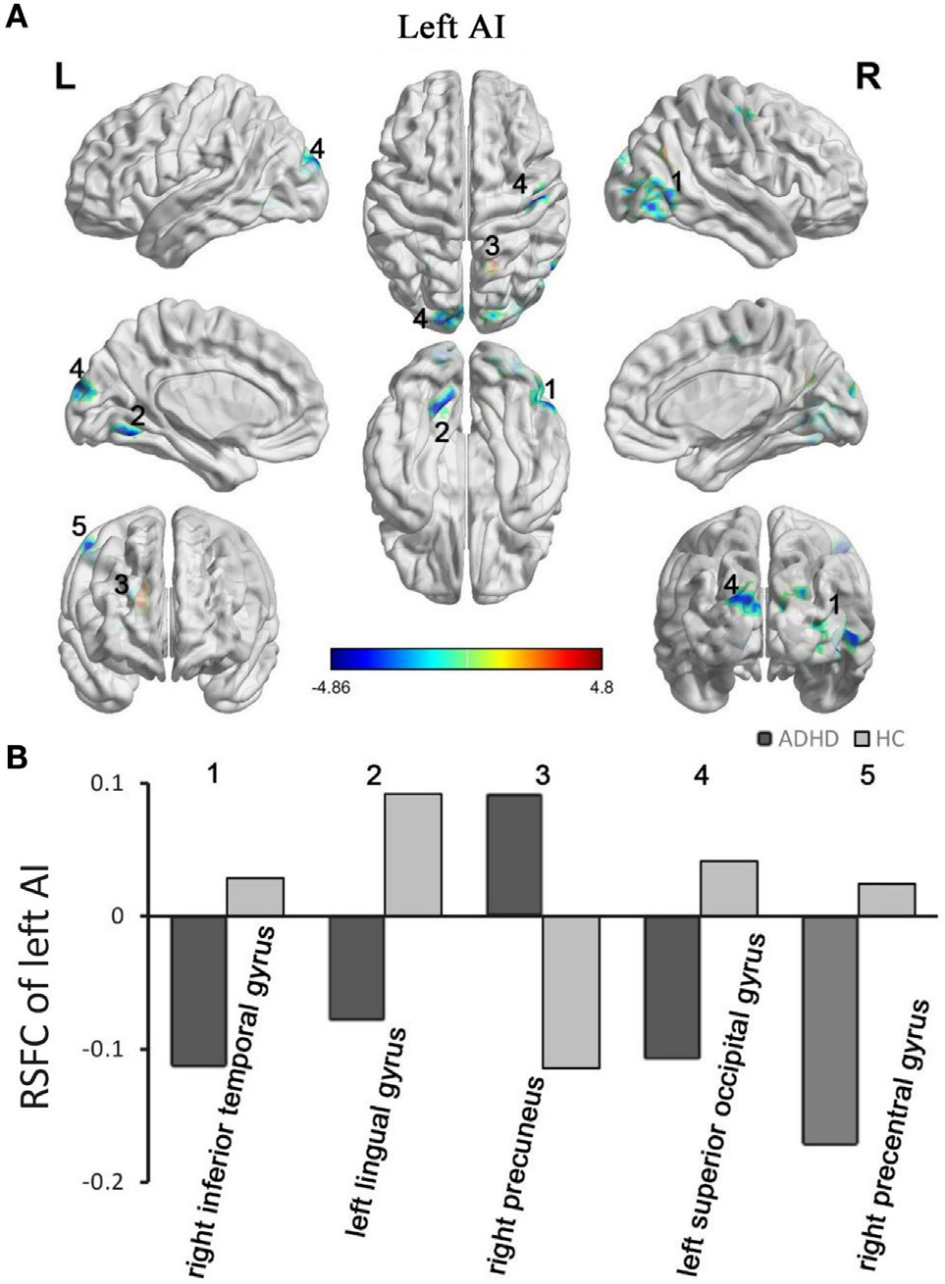
Group differences in RSFC of the left AI. Yellow color (ADHD > HC) indicates an increased functional connectivity with the insular subregions in the ADHD group, and blue color (ADHD < HC) indicates a decreased functional connectivity. **(A)** The clusters show significant differences in the RSFC of the left AI between the two groups. **(B)** Mean functional connectivity *z*-scores in clusters showing significant differences in the RSFC between the ADHD and HC groups. Numbers 1–5 marked in **(A)** refer to the brain regions in **(B)**. RSFC, resting-state functional connectivity; AI, anterior insula; HC, healthy control; ADHD, attention deficit/hyperactivity disorder.

The between-group RSFC with the left AI showed that the left AI had a positive RSFC with the right precuneus in adults with ADHD and a negative RSFC with the right precuneus in those in the HC group. The two groups showed different RSFC direction of the left AI in the right temporal gyrus, left lingual gyrus, left superior occipital gyrus, and right precentral gyrus. Specifically, the HC group displayed a positive RSFC and the patients with ADHD showed a negative RSFC between the AI and these brain regions shown above (Figure 2).

**Figure 3:**
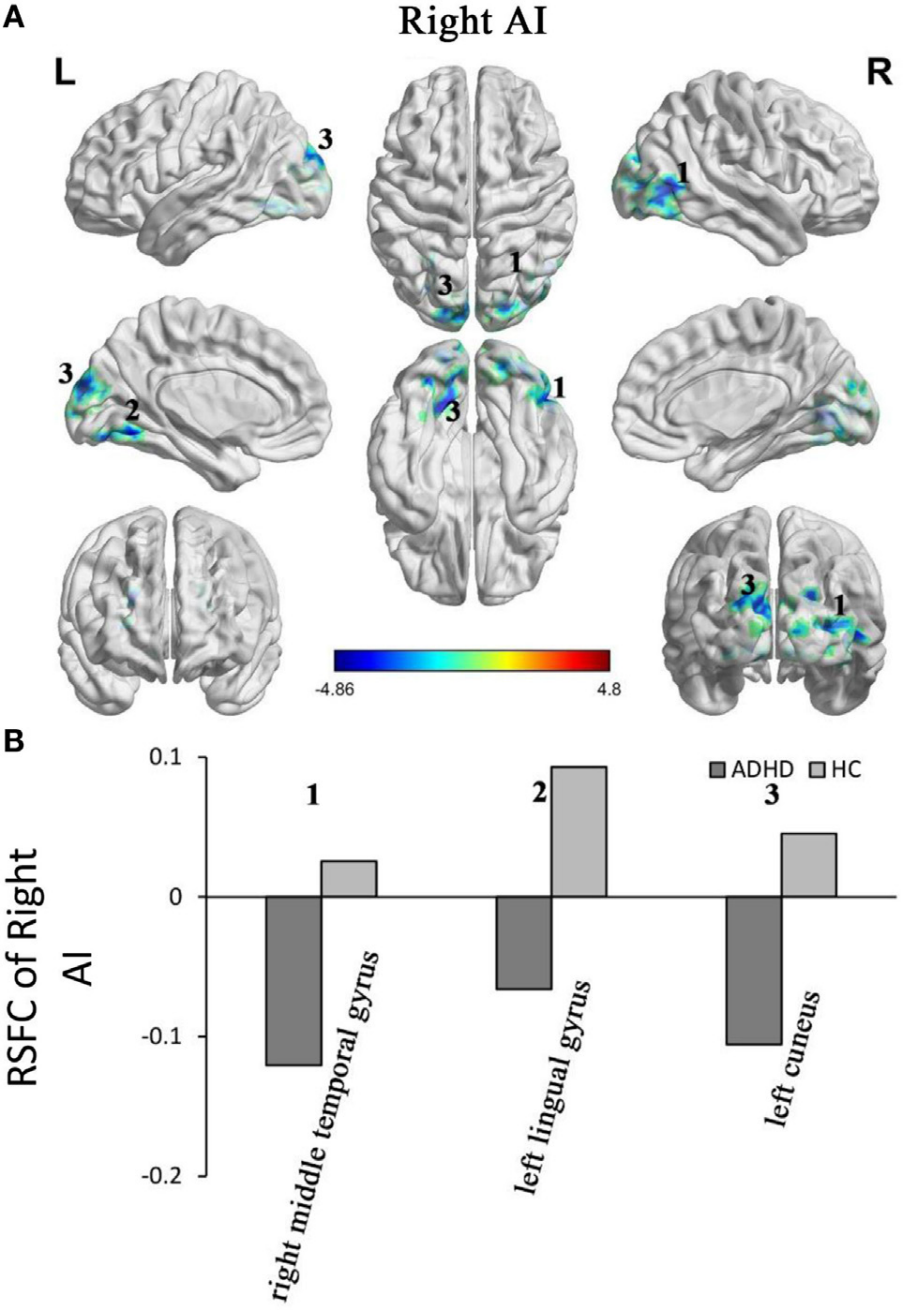
Group differences in the RSFC of the right AI. Yellow color (ADHD > HC) indicates an increased functional connectivity with the insular subregions in the ADHD group and blue color (ADHD < HC) indicates a decreased functional connectivity. **(A)** The clusters show significant differences in the RSFC of the left AI between the two groups. **(B)** Mean functional connectivity *z*-scores in clusters showing significant differences in the RSFC between the ADHD and HC groups. Numbers 1–5 marked in **(A)** refer to the brain regions in **(B)**. RSFC, resting-state functional connectivity; AI, anterior insula; HC, healthy control; ADHD, attention deficit/hyperactivity disorder.

Adults with ADHD showed negative RSFC of the right AI with right middle temporal gyrus, left cuneus, and left lingual gyrus. However, the HCs showed positive RSFC with right AI in those regions (Figure 3).

The results without GSR showed similar with results with GSR in RSFC of AI. Without GSR, bilateral AI with right precuneus showed altered RSFC in adults with ADHD. Patients with ADHD showed positive correlation and HCs showed negative correlation (Table S1 and Figures S2–S4 in Supplementary Material).

#### MI and PI

The RSFC of the other 4 ROIs, including those of the bilateral MI and PI, displayed no significant difference between the two groups in the results with GSR. There were significant differences between two groups within the results without GSR. Decreased RSFC were detected in adults with ADHD of right MI with right precuneus, left PI with right mid cingulate gyrus, right PI with right inferior temporal gyrus, right PI with right cerebellum and right PI with orbital frontal cortex (Table S1 and Figures S2–S4 in Supplementary Material).

### Correlations with the Ecological EF Scores

The RSFC *z*-values of the brain regions showing significant differences between the two groups were used for further correlation analyses with the shifting, working memory and inhibition factor scores from the BRIEF-A. In the subjects in the HC group which only 27 participants were calculated the correlations, working memory scores were positively associated with the RSFC between the left AI and right precuneus (*r* = 0.557, *p* = 0.003). The RSFC of the left AI and right inferior temporal gyrus (*r* = −0.449, *p* = 0.032) and that of the left superior occipital gyrus (*r* = −0.512, *p* = 0.006) were negatively associated with the working memory scores. The working memory scores also showed a significant negative correlation with the RSFC of the right AI and left cuneus (*r* = −0.455, *p* = 0.017). However, there was no correlation between these variables in the ADHD group.

## Discussion

This is the first study on the RSFC of the insular subregions and their correlations with ecological EF performance in adults with ADHD. First, compared with the HCs, adults with ADHD displayed general patterns of widespread altered RSFC between the AI and precuneus, occipital cortex, temporal cortex, lingual gyrus, and cuneus in the results with and without GSR. Second, within the HCs, the working memory scores were correlated with the RSFC of the bilateral AI with other brain regions. There were no significant differences of the RSFC of MI and PI in the results with GSR. However, in the results without GSR, patients with ADHD showed decreased positive functional connectivity in precuneus, temporal gyrus, cerebellum with MI and PI compared with HCs. These findings suggest that the overlapped altered functional connectivity of the AI and regions in the DMN in adults with ADHD in with GSR and without GSR may be involved in the aberrant EFs during daily life.

Altered RSFC between the left AI and right precuneus and between the bilateral AI and right medial temporal gyrus extended to the inferior temporal gyrus were observed in adults with ADHD compared with the HCs. Besides, the RSFC AI and right precuneus overlapped in the results with GSR and without GSR. The precuneus and medial temporal gyrus are two critical nodes of the DMN (60, 61) that exhibited consistently found abnormal connectivity and structure in patients with ADHD (10, 62–68). rs-fMRI studies have found decreased negative functional connectivity between the ACC, putamen, and regions in the DMN in ADHD patients (62, 67). According the previous studies, the precuneus is involved in episodic memory retrieval and self-centered mental imagery strategies (69, 70). The temporal lobe plays an important role in mnemonic processes (71). The DMN is involved in internal and external mental processing, such as attention, episodic memory retrieval, envisioning the future, and decision making (71). The abnormal RSFC between the key nodes of the SN and the DMN in patients with ADHD provides support for our prediction. The AI is involved in salience stimuli allocation and DMN disengagement, which play an important role in attention, working memory and higher order cognition (15, 22), and the altered RSFC may suggest that the AI fails to disengage the DMN or disengage it by mistake. Patients with ADHD are often easily distracted by unrelated stimuli (internal or external), which might indicate a misallocation of salience by the AI. This aberrant process uncovered by our results might lead to the EF defects. The results are also in line with recent resting-state studies examining SN abnormality in patients with ADHD (16, 72). Another study found that the improvement of ADHD symptom improvement in adults with ADHD by taking in amphetamine was linked to altered insula-medial prefrontal cortex (a core part of DMN) connectivity (73). These consistent results may suggest that the abnormal salience stimuli allocation procedure mediated by the AI, might be involved in the cognitive and EFs. We supposed that the impairment of the process in which the AI distributes different salient stimuli (internal or external) potentially results in the different subtypes of ADHD. Future studies are needed to verify this hypothesis.

We still identified an altered RSFC of the bilateral AI with left lingual gyrus. The lingual gyrus participates in visual phonological and word processing (74, 75). rs-fMRI and event-related fMRI studies have observed a lingual gyrus abnormality in patients with ADHD (26). Ko et al. identified a higher activation in the lingual area during a working memory dual task, which supports our findings that the lingual gyrus may involve in working memory process. The altered RSFC of bilateral AI with left lingual gyrus did not detect in the results without GSR in patients with ADHD. Future studies should therefore include the reason leading to the inconsistent results with and without GSR.

Our findings identified that the working memory factor scores were positively associated with the RSFC between the left AI and precuneus and that they were negatively correlated with the left AI and right temporal lobe in the HC group. The correlation between the working memory scores and the RSFC of the right AI and right temporal lobe exist with a tendency (*p* = 0.062). There was no correlation of these variables in the ADHD group. The results identified that participants with a higher RSFC (positive and negative) had better working memory performance during daily life in the HC group. However, these associations between working memory and the RSFC were disrupted in ADHD group. The disrupted correlation might influence the working memory procedure and therefore suggest working memory impairment. Based on the previous studies (15, 22), we speculate that salient stimuli were transferred to the AI and that the AI conveyed altered control signals to other large-scale networks related to working memory resources, involved in the working memory impairment in patients with ADHD.

We also found the altered direction in the RSFC of the left AI with the right precentral gyrus in adults with ADHD compared with the HCs. The precentral gyrus, which is implicated in multiple EFs, such as sustained attention, working memory and response inhibition, and task switching is part of the motor cortex (24, 76–78). We supposed that the abnormal allocation of salient stimuli in the AI is involved an aberrant bottom-up control of the precentral gyrus and then results in attention or EF symptoms. However, there was no correlation between the EF factor scores and the RSFC of the left AI with the right precentral in either of the groups. This discrepancy may be due to differences the experimental conditions (“resting-state” vs. “inhibiting task” or “task switching task”).

We did not identify differences in the RSFC of the MI and PI with the other brain regions between the two groups in the results with GSR. And there were some differences between two groups in the results without GSR. These results may suggest that AI which had overlapped results in two methods deficits play a major role in the ADHD mechanism. However, future studies are needed to replicate these results.

Several studies have raised concerns regarding the anticorrelated networks after GSR (52, 79), and others have deemed the anticorrelated network a true neural signal (80). GSR generally affects between-group analyses in complicated ways. In this situation, there will be distinct results between the with GSR and without GSR analyses. We completed another analysis without GSR, and the between-group difference patterns were altered without GSR. There are some differences between the two groups in the RSFC of the insular subregions (Table S1 and Figures S2–S4 in Supplementary Material). Consistent with previous studies with GSR that found an anticorrelation with the precuneus or posterior cingulate gyrus (63, 67), we found an anticorrelation of the AI and precuneus in the HCs. As shown in the figure, the anticorrelation between the AI and precuneus showed in the results after GSR decreased without GSR. Therefore, our findings should be interpreted with caution.

There are several limitations of this study that should be considered. First, our participants included patients with two subtypes of ADHD (ADHD-I and ADHD-C). To explore fully how the ADHD subtypes moderate such relationships, a separate study with a much larger sample is needed. Furthermore, the IQs of the participants of the two groups in our study were high. Though the IQs of the two groups had no significant statistical differences, a high IQ may cover up some other differences in adults with ADHD. Next, 6 of the 28 participants in the ADHD group had received medication treatment before the assessment. However, after excluding these six patients, the results did not change substantially and our conclusion was not influenced. Finally, the results of the without GSR analyses (Table S1 and Figures S2–S4 in Supplementary Material) showed different pattern with the results using GSR. Therefore, our findings should be interpreted with some caution and need further replication with regard to the abovementioned limitations.

In summary, this study was the first to detect the abnormality of the insular subregions’ functional networks among adults with ADHD and determined that the EF factor scores are associated with the RSFC of the AI with the precuneus and temporal gyrus in the HC group. However, there is no correlation of the EF factor and with the RSFC of the AI in the ADHD group. The normal correlations between the working memory and RSFC of the AI were disrupted in adults with ADHD. The results indicate that disrupted SN-DMN networks might be involved in EF impairment in adults with ADHD, especially working memory. Our findings suggest that deficits of AI particularly, which refer to abnormal salient stimuli allocation, might be involved in working memory impairment in patients with ADHD. The inconsistent results of MI and PI between results with and without GSR need further exploration.

## Ethics Statement

This study was approved by the Research Ethics Review Board of Peking University Sixth Hospital. All subjects were fully informed about the research before being asked to sign the informed consent form.

## Author Contributions

QZ and LS contributed to the design of the work. QZ, HL, XY, FH, YW, QQ, LS, LL, and YW were involved in the acquisition of the data. QZ, HL, XY, and LS participated in the analysis and interpretation of the data. QZ and LS drafted the work. LL, YZ, QC, and YW revised it critically.

## Conflict of Interest Statement

The authors declare that the research was conducted in the absence of any commercial or financial relationships that could be construed as a potential conflict of interest.
