# Supplementary material for: Abnormal Resting-State Functional Connectivity of Insular Subregions and Disrupted Correlation with Working Memory in Adults with Attention Deficit/Hyperactivity Disorder
Source: Front Psychiatry. 2017 Oct 11;8:200. doi: 10.3389/fpsyt.2017.00200 (PMC5641567; doi:10.3389/fpsyt.2017.00200)

Supplementary Materials

| Table S1. Clusters Displaying Significant Group Differences in RSFC of insular subregions between the ADHD and HD groups (without global signal regression) | | | | | | | |
| --- | --- | --- | --- | --- | --- | --- | --- |
| Seed | Area | L/R | Cluster  size | x | MNI  y | z | *t* value |
| Left AI |  |  |  |  |  |  |  |
|  | Precuneus | R | 47 | 18 | -57 | 24 | 5.090 |
| Right AI |  |  |  |  |  |  |  |
|  | Precuneus | R | 238 | 18 | -57 | 24 | 4.796 |
| Right MI |  |  |  |  |  |  |  |
|  | Precuneus | R | 1751 | 15 | -66 | 30 | 5.159 |
| Left PI |  |  |  |  |  |  |  |
|  | Mid cingulate gyrus | R | 70 | 3 | -30 | 45 | 3.850 |
| Right PI |  |  |  |  |  |  |  |
|  | Inferior temporal gyrus | R | 8981 | 60 | -18 | -27 | 4.575 |
|  | Cerebellum | R | 47 | 6 | -75 | -42 | 4.817 |
|  | orbital frontal cortex | R | 640 | 6 | 63 | -6 | 3.8185 |
| Note: MNI = Montreal Neurological Institute. AI = anterior insula; MI = mid insula; PI = posterior insula; L = left; R = right; HC = healthy control | | | | | | | |
| Table S2. Clusters Displaying Significant Group Differences in RSFC of insular subregions between the ADHD and HD groups(using insular subregion masks from Kelly et., al (Kelly et al., 2012) | | | | | | | |
| Seed | Area | L/R | Cluster  size | x | MNI  y | z | *t* value |
| Left AI |  |  |  |  |  |  |  |
|  | Superior occipital gyrus | R | 782 | 24 | -90 | 12 | -4.331 |
|  | Superior occipital gyrus | L | 194 | -15 | -84 | 24 | -4.072 |
|  | Precuneus | R | 82 | 18 | -54 | 24 | 5.204 |
|  | Postcentral gyrus | R | 139 | 27 | -39 | 72 | -4.979 |
| Right AI |  |  |  |  |  |  |  |
|  | Superior occipital gyrus | L | 559 | -15 | -84 | 24 | -4.245 |
| Left MI |  |  |  |  |  |  |  |
|  | Calcarine | R | 390 | 9 | -93 | -3 | -4.246 |
|  | Superior occipital gyrus | L | 202 | -15 | -84 | 24 | -5.349 |
| Note: MNI = Montreal Neurological Institute. AI = anterior insula; MI = mid insula L = left; R=right; HC = healthy control | | | | | | | |


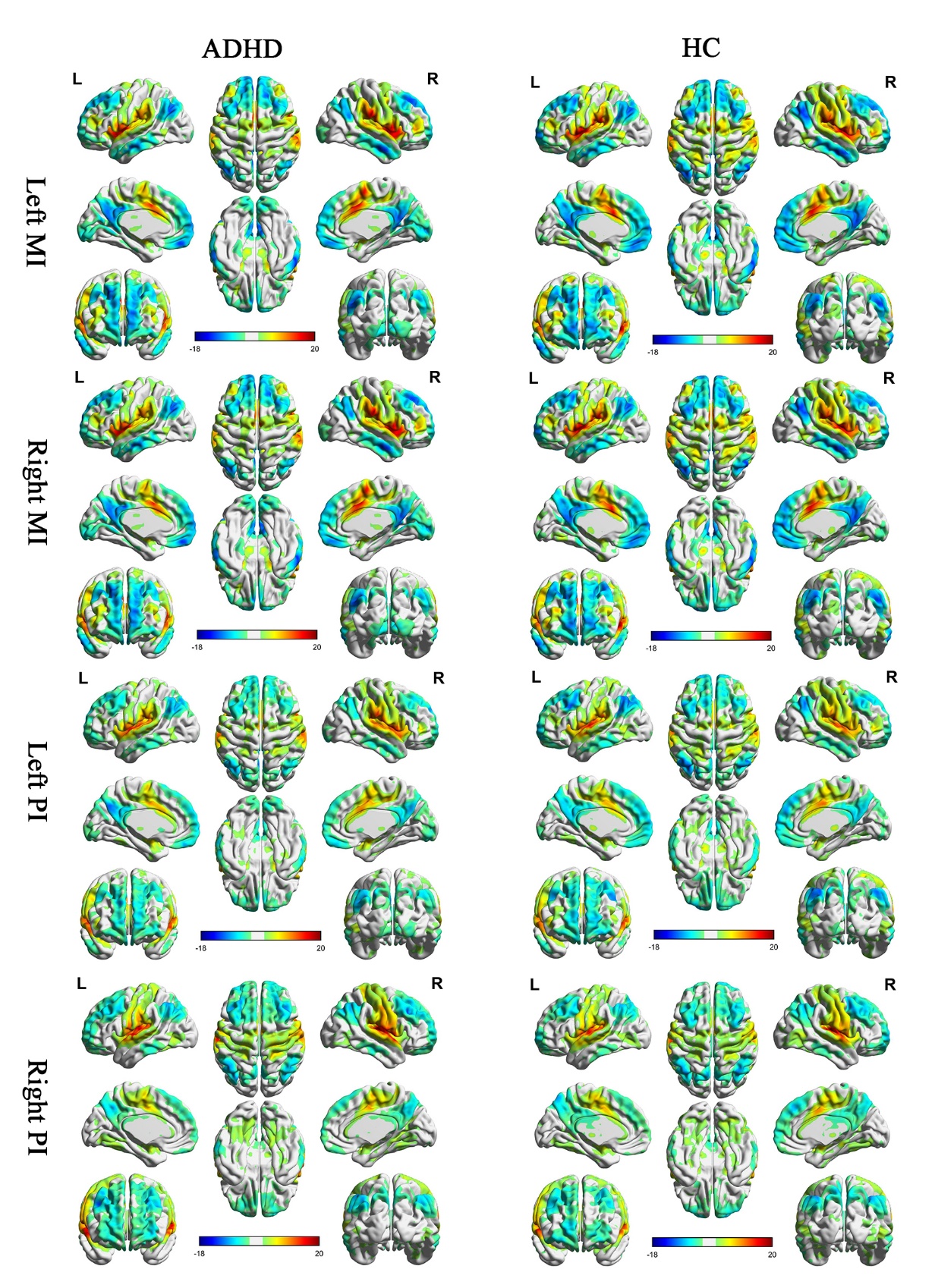


Figure S1 Significant RSFC patterns of bilateral mid and posterior insula in the ADHD and HC groups. The yellow red colors suggest the brain regions were positively correlated with insular subregions and the blue colors suggest negative correlations with insular subregions. MI = mid insular; PI = posterior insula; HC = healthy control

Figure S2 Group differences in RSFC of the bilateral AI and right MI. Yellow color (ADHD > HC) indicates an increased functional connectivity with the insular subregions in the ADHD group and blue color(ADHD<HC) indicates a decreased functional connectivity. The clusters show significant differences between two groups in RSFC of left AI. RSFC= resting state functional connectivity; AI = anterior insula. MI = mid insula HC = healthy controls.


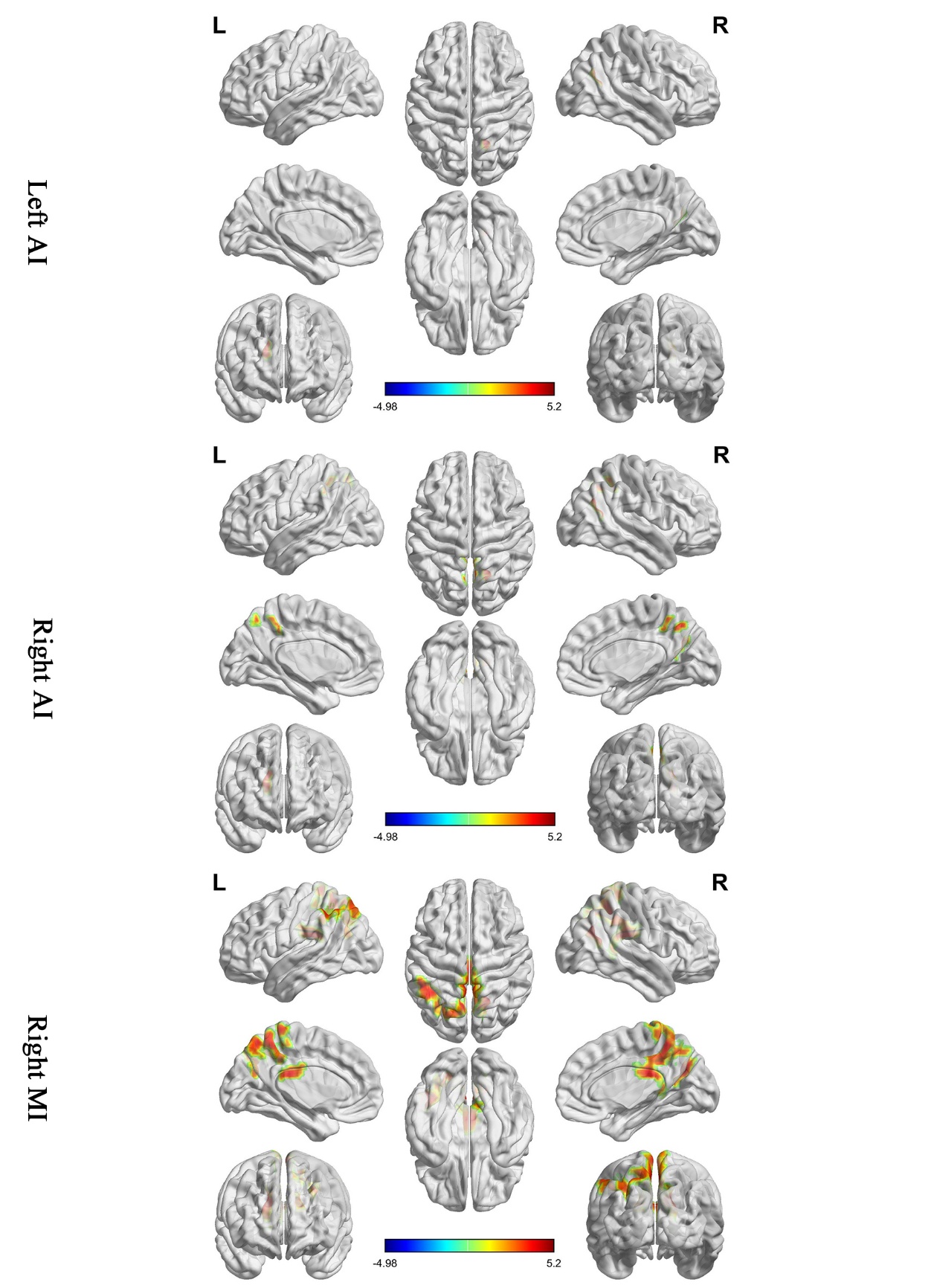


Figure S3 Group differences in RSFC of the bilateral PI. Yellow color (ADHD > HC) indicates an increased functional connectivity with the insular subregions in the ADHD group and blue color(ADHD < HC) indicates a decreased functional connectivity. The clusters show significant differences between two groups in RSFC of left bilateral PI. RSFC= resting state functional connectivity; PI = posterior insula. HC = healthy control.


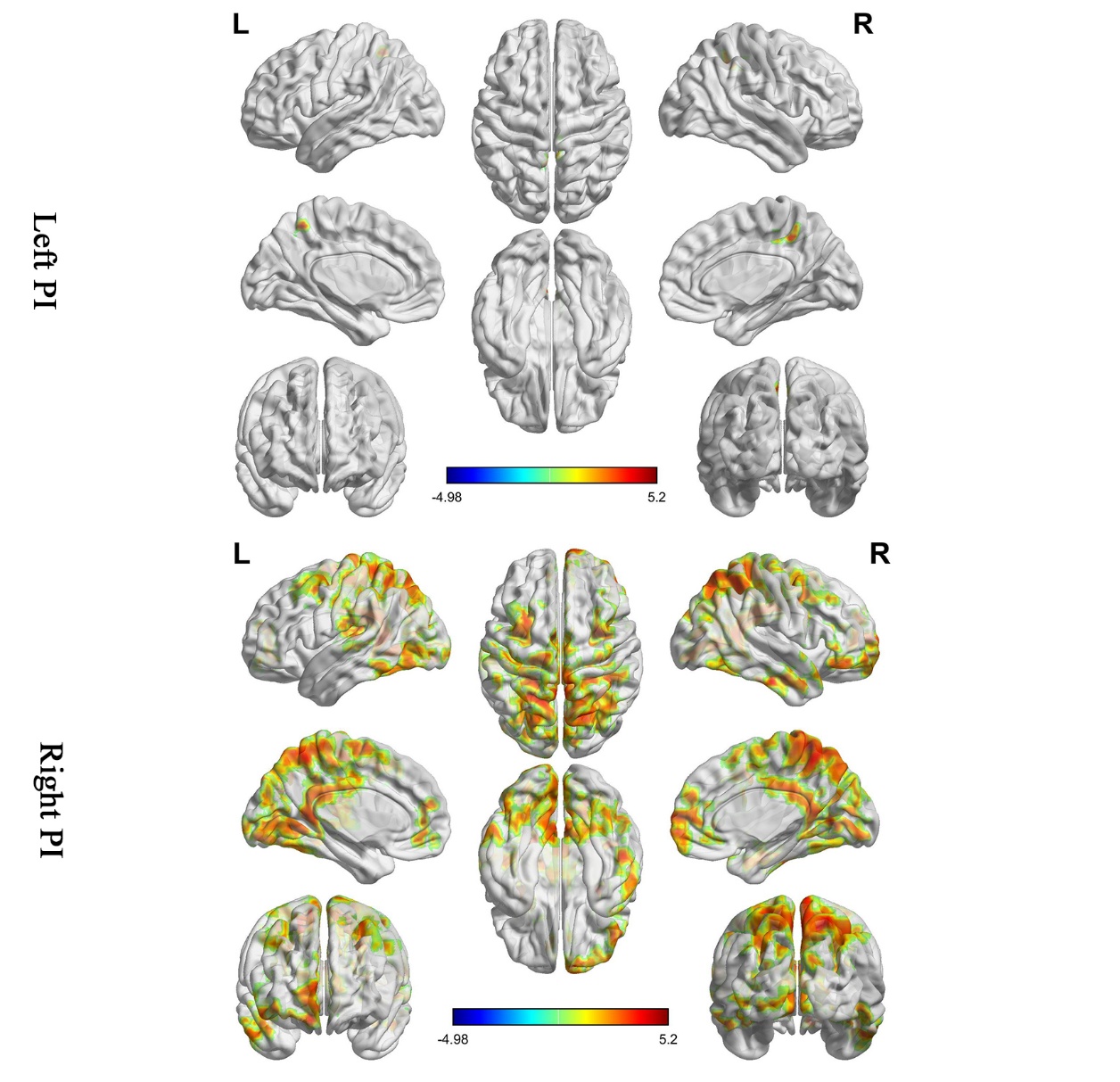


Figure S4 Mean functional connectivity z-scores in clusters showing significant differences in RSFC for the ADHD and HC group. RSFC = resting state functional connectivity; AI = anterior insula; MI = mid insula; PI = posterior insula HC = healthy control.


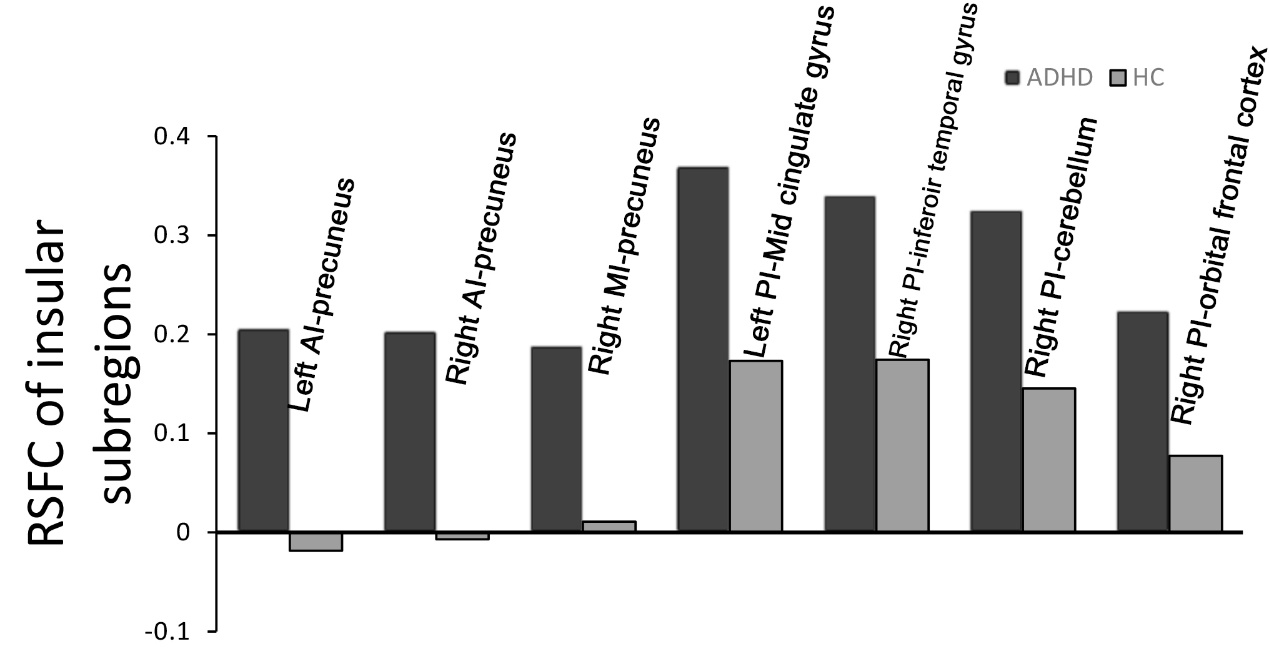


Figure S5 Group differences in RSFC of the bilateral AI and left MI using insular subregions masks and spherical ROIs. Yellow color (ADHD > HC) indicates an increased functional connectivity with the insular subregions in the ADHD group and blue color (ADHD < HC) indicates a decreased functional connectivity. The clusters show significant differences between two groups in RSFC of left bilateral PI. RSFC = resting state functional connectivity; AI=anterior insula. MI = mid insula HC = healthy control.


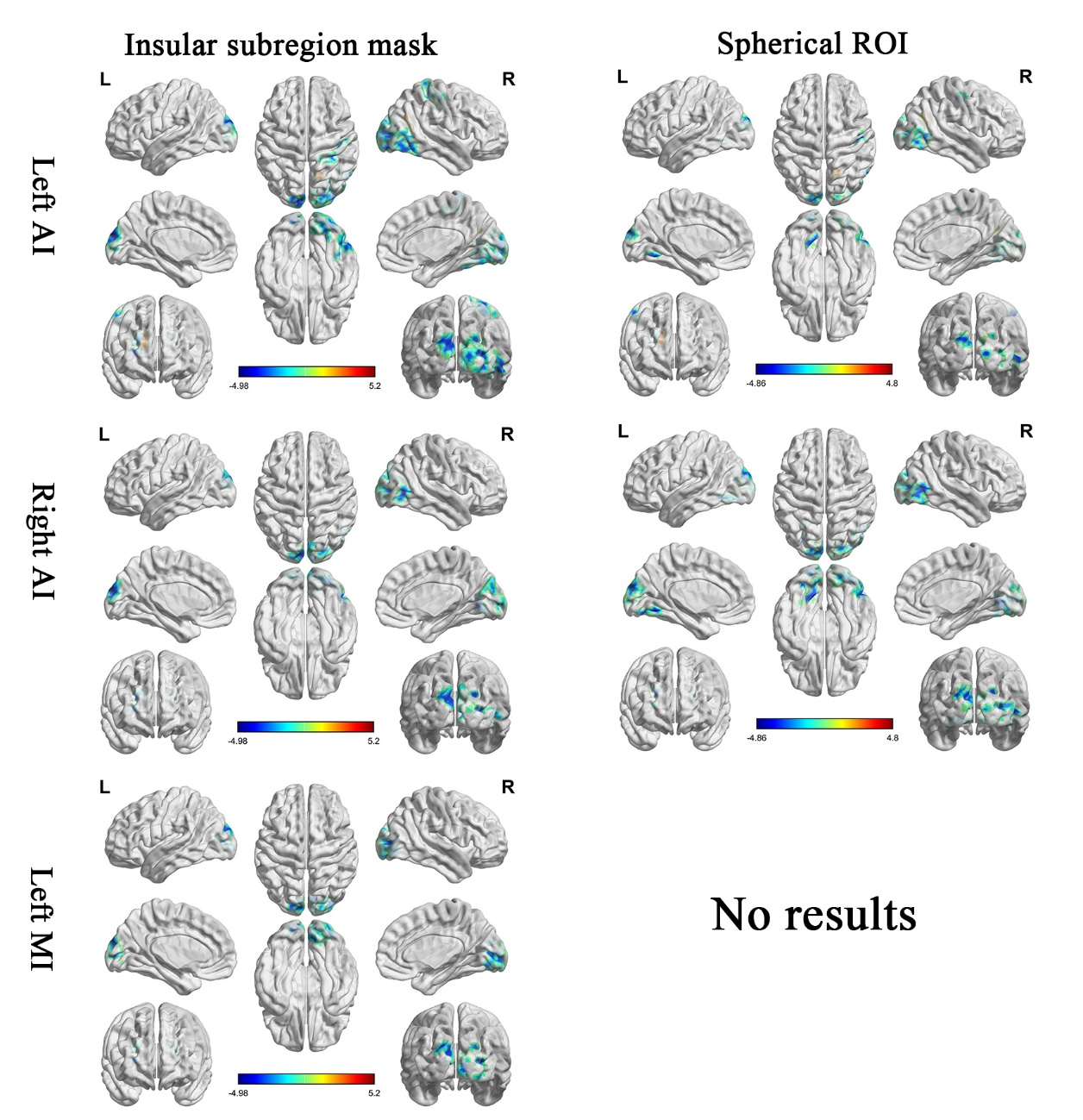

Supplement: Supplementary file 1 [file data_sheet_1.docx]
